# Dahl's Sign: An Indicator of Severe Chronic Obstructive Pulmonary Disease

**DOI:** 10.7759/cureus.6865

**Published:** 2020-02-04

**Authors:** Sreenath Meegada, Madhavi Annakula, Tejo Challa, Suman Siddamreddy, Prashanth Peddi

**Affiliations:** 1 Internal Medicine, The University of Texas Health Science Center/Christus Good Shepherd Medical Center, Longview, USA; 2 Internal Medicine, Baptist Health Medical Center, North Little Rock, USA

**Keywords:** dahl's sign, thinker's sign, copd, severe chronic respiratory disease

## Abstract

Dahl’s sign, a clinical sign in which areas of thickened and darkened skin seen on the lower thighs and/or elbows, is seen in patients with severe chronic respiratory disorders such as chronic obstructive pulmonary disease (COPD), interstitial lung disease, congestive heart failure (CHF), and chronic moderate to severe persistent asthma. The aim of our present report is to create awareness and encourage providers to lay emphasis on physical examination in every medical examination that can give clues to the severity of the underlying disorder.

## Introduction

Chronic obstructive pulmonary disease (COPD) is a global health problem that affects approximately 251 million people worldwide. Tobacco use is the most common cause of COPD. The diagnosis is primarily based on spirometry. Barrel chest, pursed-lip breathing, and clubbing are well-recognized findings among COPD patients. Dahl’s sign or Thinker’s sign, a clinical sign in which areas of thickened and darkened skin are seen on the lower thighs and/or elbows, was described as early as 1963 but is not commonly recognized among students and training physicians [1]. There is no documentation in the literature regarding the incidence of Dahl's sign in COPD. Creating awareness of this important physical exam finding will aid many physicians to diagnose severe COPD at the bedside.

## Case presentation

A 67-year-old African American male with a history of COPD on home oxygen 4 liters per minute, hypertension, hyperlipidemia, ongoing tobacco abuse one pack per day presents with cough, shortness of breath, and wheezing for three days. The cough was productive with yellowish, white sputum and increased volume. He had dyspnea at rest, which is worse with minimal exertion. He denies any orthopnea, paroxysmal nocturnal dyspnea, swelling of legs, and weight gain. He complains of chest pain with every coughing spell. Chest pain is located in the parasternal area, aggravated with cough or deep breaths, non-radiating, not associated with diaphoresis or palpitations. He denied any fevers, chills, or rigors and had no sick contacts. He tried albuterol nebulization at home with minimal improvement in dyspnea.

**Figure 1:**
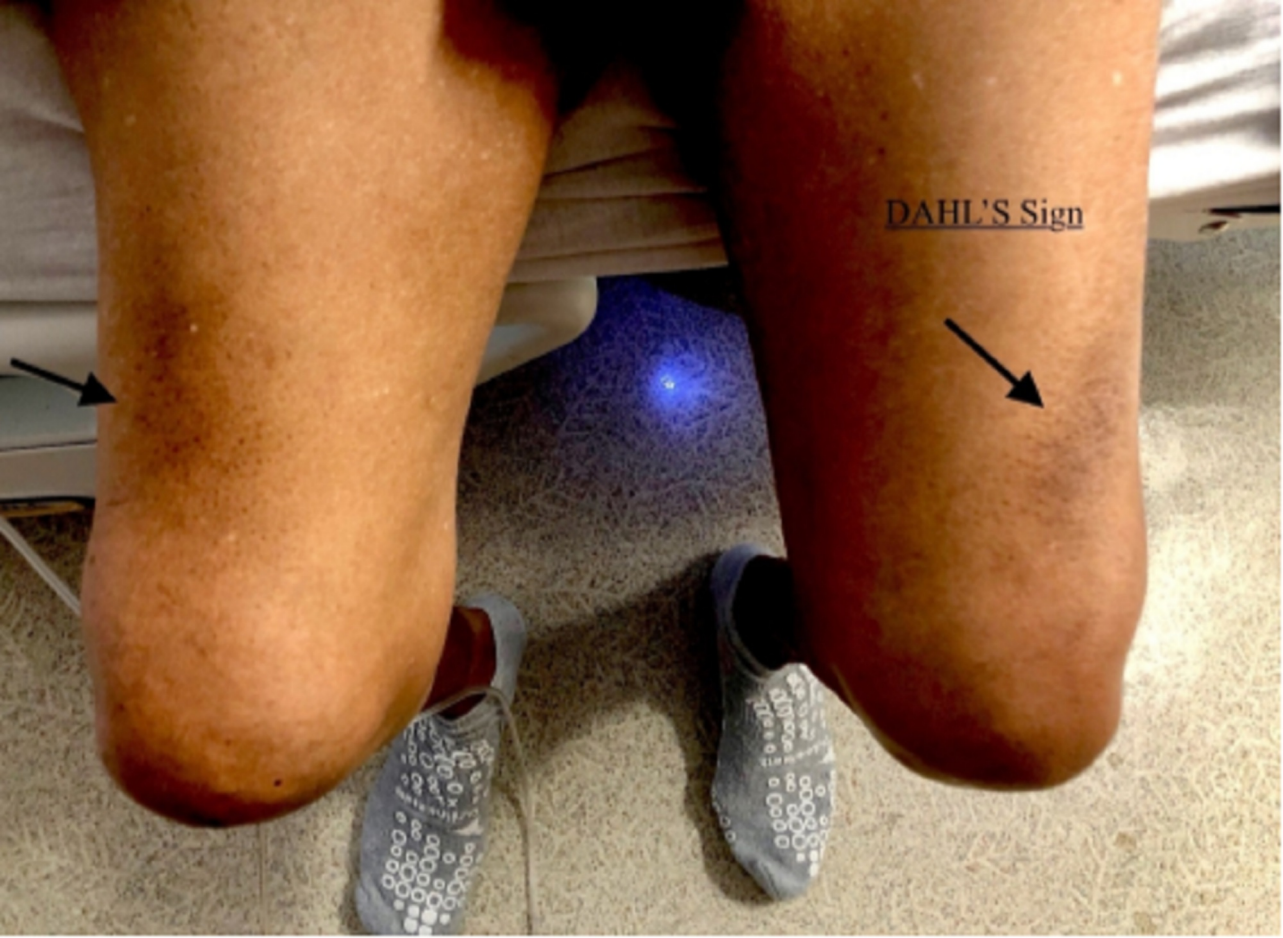
Hyperpigmented skin patches on the lower third of both thighs (arrows) - Dahl's sign

In the emergency room, he was found to be in moderate to severe respiratory distress, with a respiratory rate of 28 per minute, oxygen saturation of 77% on room air, tachycardia with a heart rate of 98, in labored breathing, using the accessory muscles of the neck, and in tripod position with his hands on the lower part of the thighs. Auscultation of lungs demonstrated bilateral wheezing with intermittent rhonchi. There were normal heart sounds with no murmurs or gallops on the heart exam. Skin examination showed symmetric hyperpigmented and hyperkeratotic areas on the lower part of both thighs - Dahl's sign (Figure 1).

He minimally responded to steroids, nebulizer treatments with albuterol and ipratropium, and supplemental oxygen, after which he was started on noninvasive ventilation, i.e. bilevel positive airway pressure (BiPAP) and transferred to intensive care unit (ICU) for further care.

Initial labs including complete blood cell count was normal except for a low hemoglobin of 11.0 gram per deciliter, metabolic panel was normal, arterial blood gas was abnormal with a low pH of 7.2, high partial pressure of carbon dioxide (pCO2) of 60, low pO2 of 48 prior to bilevel positive airway pressure (BiPAP) placement, chest X-ray showed hyperinflation findings consistent with COPD, and no acute infiltrate. In the intensive care unit (ICU), he was continued on BiPAP for two days with good improvement in arterial blood gases. The patient was weaned to oxygen at 4 liters per minute. He was downgraded to the regular floor after two days of treatment in the ICU.

The patient significantly improved after two days of treatments, returned to his baseline, and advised to follow-up with the primary care physician and pulmonologist. He was discharged home on a long-acting anti-cholinergic inhaling agent (tiotropium bromide), long-acting beta-agonist/inhaled steroids twice daily (formoterol/budesonide), and rescue inhalers (albuterol or ipratropium) as needed, and resumed home oxygen at 4 liters/minute. He was strictly counseled regarding smoking cessation prior to discharge. He had pulmonary function testing (PFT) done at the pulmonologist's office, which showed a forced expiratory volume (FEV1) of 18%.

## Discussion

Dahl’s sign or Thinker’s sign was first described by Rothenberg HJ and Dahl MV in 1963 and 1970 [1-2]. This sign is classically seen on the lower part of the thighs of patients who spend a lot of time in the tripod position with elbows or hands resting on their thighs [1]. Hyperpigmentation is caused by chronic pressure or irritation on the skin epidermis from the elbows or hands of patients for a longer duration of time in the tripod position. Chronic intermittent pressure induced by the friction of the elbows or hands on the thighs will result in the release of hemosiderin from erythrocytes and the proliferation of the stratum corneum of the skin, resulting in Dahl’s sign [3].

Dahl’s sign is seen in patients with conditions causing chronic respiratory distress such as COPD, interstitial lung disease, bronchial asthma, and congestive heart failure [4-5]. The classic tripod position optimizes the inspiratory accessory muscles’ effort, lets the diaphragm get into its natural shape, and has a piston-like mechanism to help inspiration, which, in turn, relieves dyspnea [6].

Dahl’s sign is a predictor of the severity of the underlying respiratory disorder, in this case, COPD. It is observed in patients with severe COPD, i.e FEV1 less than 30% [5]. Our patient had an FEV1 of 18%, which falls into the category of severe COPD.

## Conclusions

Dahl’s sign emphasizes a comprehensive physical examination along with a clear and detailed history to be an essential part of any medical assessment. Dahl’s sign provides a clinical marker of lung hyperinflation and deconditioning that should alert the practitioner in charge of the extreme severity of the chronic respiratory disease. Tripoding (bracing of arms on both legs) will improve respiratory mechanics and improve dyspnea in patients with COPD. Barrel chest, pursed-lip breathing, and hypertrophied sternomastoid are the other clues for severe COPD.
